# Tissue- and compartment-resolved brain age reveals vascular-metabolic and neurodegenerative aging

**DOI:** 10.21203/rs.3.rs-10808325/v1

**Published:** 2026-09-11

**Authors:** Hosung Kim, Cong Zang, Jinsan Lee, Elizabeth Haddad, Gilsoon Park, Jianwei Zhang, Jahae Kim, Kangho Choi, Yaqiong Chai, Andrei Irimia, Priya Rajagopalan, Won-Jin Moon, Yonggang Shi, Hyung-Jeong Yang, Arthur Toga, Neda Jahanshad

**Affiliations:** USC Stevens Neuroimaging and Informatics Institute, Keck School of Medicine of USC, University of Southern California; USC Stevens Neuroimaging and Informatics Institute, Keck School of Medicine of USC, University of Southern California; Department of Neurology, Kyung Hee University Hospital, Kyung Hee University College of Medicine; USC Stevens Neuroimaging and Informatics Institute, Keck School of Medicine of USC, University of Southern California; USC Stevens Neuroimaging and Informatics Institute, Keck School of Medicine of USC, University of Southern California; USC Stevens Neuroimaging and Informatics Institute, Keck School of Medicine of USC, University of Southern California; Department of Nuclear Medicine, Chonnam National University Hospital; Department of Neurology, Chonnam National University Hospital; USC Stevens Neuroimaging and Informatics Institute, Keck School of Medicine of USC, University of Southern California; Ethel Percy Andrus Gerontology Center, Leonard Davis School of Gerontology; Los Angeles Medical Center; Department of Radiology, Konkuk University Medical Center; USC Stevens Neuroimaging and Informatics Institute, Keck School of Medicine of USC, University of Southern California; Department of Artificial Intelligence Convergence, Chonnam National University; Laboratory of NeuroImaging, USC Stevens Neuroimaging and Informatics Institute, Keck School of Medicine, University of Southern California; USC Stevens Neuroimaging and Informatics Institute, Keck School of Medicine of USC, University of Southern California

**Keywords:** Brain age prediction, white matter aging, T2-FLAIR MRI, cardiometabolic risk, Alzheimer’s disease, amyloid and tau PET, multimodal neuroimaging

## Abstract

Brain aging reflects heterogeneous vascular-metabolic and neurodegenerative processes, yet brain age is often reduced to a gray matter (GM)-dominant estimate. Emerging evidence suggests that subtle “dirty-appearing” T2-FLAIR abnormalities may mark diffuse white matter (WM) vulnerability that precedes or extends beyond overt WM hyperintensities (WMH). Here we developed a compartment-resolved framework integrating T1-weighted GM features with unthresholded T2-FLAIR WM signal across depth- and lobe-specific surfaces. In 26,446 UK Biobank and 992 ADNI participants, WM brain age captured variation beyond WMH burden and was preferentially associated with cardiometabolic risk before age 65; periventricular WM age statistically linked cardiometabolic burden to cognition. Across the Alzheimer’s disease continuum, GM and WM brain age increased with diagnostic severity but showed distinct molecular associations: GM aging aligned more closely with amyloid burden, whereas WM aging showed prominent tau associations. These findings reveal complementary vascular-metabolic and neurodegenerative dimensions of brain aging obscured by global brain age estimates.

Human brain aging is profoundly heterogeneous such that individuals of the same chronological age can differ markedly in neural integrity, cognitive resilience and vulnerability to neurodegenerative disease^1^. Machine learning can be used to predict an individual’s apparent brain age from distributed neuroimaging features, thereby providing a quantitative index of deviation from normative aging trajectories^2,3^. The resulting brain age gap (BAG), defined as the difference between the predicted brain age and chronological age, is commonly interpreted as a marker of accelerated or resilient brain aging and has been associated with cognitive decline, vascular and metabolic risk, and neurodegenerative disease^4–6^.

Despite its growing relevance, brain age research has remained largely gray matter-centric, relying predominantly on T1-weighted MRI measures of cortical thickness, volume and morphology^3–5^. This emphasis on gray matter (GM) captures a critical but incomplete view of brain aging. Cortical atrophy albeit linked closely to neurodegeneration and cognitive impairment, may only reflect a relatively late-stage of structural damage^7^. By contrast, white matter (WM), is highly vulnerable to vascular, metabolic and microstructural injury which may precede overt cortical loss^8,9^. Conventional WM analyses have focused mainly on WM hyperintensity (WMH) burden measured from T2-FLAIR MRI, a clinically established marker of cerebral small vessel disease^10^. However, WMH represents only the visible end of a broader pathological spectrum^11^. Subtle and spatially diffuse FLAIR abnormalities can occur in WM outside overt WMH, often described as *dirty-appearing* or *diffusely abnormal* white matter (DAWM)^11,12^. However, its relationship with cognitive decline and dementia remains uncertain, highlighting the complexity and biological heterogeneity of these subtle signal abnormalities^13^. Beyond visually defined WMH and DAWM, spatially distributed variation in T2-FLAIR signal across normal-appearing WM may reflect a composite of age-related tissue changes, including vascular injury, myelin disruption, axonal injury, gliosis and altered interstitial water content^9^. Unthresholded T2-FLAIR signal intensities, rather than binary or volumetric WMH measures, may therefore provide a quantitative representation of this broader spectrum of WM alteration.

Here we tested whether WM brain age derived from unthresholded T2-FLAIR signal intensities, encompassing subtle abnormalities across normal-appearing WM as well as overt lesions, provides a complementary imaging-derived marker of brain aging to GM brain age derived from T1-weighted MRI. The conceptual advance of this study is not simply the construction of parallel GM and WM brain age models, but the evaluation of whether tissue- and compartment-resolved brain age estimates preferentially associate with distinct vascular-metabolic, cognitive and Alzheimer’s disease-related processes. This distinction is important because GM and WM are shaped by different biological processes, evolve over different temporal scales and may show distinct regional vulnerabilities to vascular burden, cognitive decline and Alzheimer’s disease (AD) pathology^6,14,15^. A single global brain age estimate may therefore obscure partially separable aging processes arising from distinct tissue compartments and anatomical systems, limiting biological interpretability and sensitivity to disease-specific mechanisms^16^.

WM aging is a heterogeneous process that varies across tissue depth, with emerging evidence indicating that distinct white matter compartments may exhibit different patterns of vulnerability to vascular and neurodegenerative processes. This is likely owing to differences among periventricular WM (pvWM), deep WM (dWM) and juxtacortical WM (jxWM) in their vascular supply, connectivity architecture and susceptibility to injury^17,18^. This compartmental organization is clinically relevant because cognitive impairment in later life commonly reflects mixed vascular and neurodegenerative contributions rather than a single pathological process. Vascular injury preferentially affects periventricular and deep WM through small-vessel disease, impaired perfusion and perivascular dysfunction, whereas juxtacortical WM lies adjacent to cortical association regions and contains short association/U-fibers that support cortico-cortical communication^10,17–19^.A depth-resolved WM brain age model may therefore help distinguish vascular-dominant WM vulnerability from WM alterations more closely coupled to cortical AD-related pathology. We therefore developed a regional WM brain age framework that combines depth-based WM partitioning with lobar anatomical organization, enabling FLAIR-derived aging estimates across distinct WM compartments. In parallel, we estimated regional GM brain age from T1-weighted MRI, enabling direct comparison of tissue-specific aging signatures across MRI modalities.

Using the UK Biobank^20^ and the Alzheimer’s Disease Neuroimaging Initiative^21^, we tested whether brain age can be resolved into tissue- and compartment-specific signatures that capture complementary vascular-metabolic and neurodegenerative dimensions of aging. We hypothesized that T2-FLAIR-derived WM brain age would exhibit stronger associations with cardiovascular and metabolic risk factors than T1-derived GM brain age, particularly within periventricular white matter regions, whereas GM brain age would show stronger associations with cognitive performance and AD pathology. We thus assessed the association of WM brain age and GM brain age with cardiovascular and metabolic risk, cognitive performance and AD molecular pathology, including amyloid-β and tau burden^22–24^. To determine whether these relationships vary across adulthood, we stratified participants into midlife adults aged <65 years and older adults aged ≥65 years. We further compared regional BAGs with their constituent imaging features to determine whether machine-learning-derived brain age provides additional sensitivity to biological and clinical variation beyond conventional structural measures alone^3,4,16^.

To address these questions, we developed a multimodal regional brain age framework that extends our previous GM brain age approach^25^ to compartment-resolved WM brain age estimation. The framework integrates functionally organized cortical regions with lobar and depth-specific WM compartments, enabling direct comparison of GM and WM aging signatures in relation to cardiometabolic burden, cognition and AD pathology (the workflow are illustrated in Fig. 1).

## Results

### Participant Characteristics.

The UK Biobank participants were late middle-aged to older adults, with a mean age of 64.7 years (s.d. 7.5), and 50.76% were male. Mean education duration was 16.54 years (s.d. 3.89). Imaging data were acquired across the three UK Biobank imaging sites: Newcastle, Cheadle and Reading. The mean interval between baseline assessment and imaging was 8.84 years (s.d. 1.73).

The UK Biobank sample showed broad variation in vascular and metabolic risk burden as seen in Table 1. Clinical diagnoses included obesity in 5,995 participants, diabetes in 2,061 participants and hypertension in 6,584 participants. Cognitive outcomes included memory, executive function, processing speed and reasoning scores, which were standardized and averaged to generate a composite cognitive score for downstream analyses.

The ADNI cohort comprises 480 cognitively normal participants, 328 participants with mild cognitive impairment and 184 participants with AD (Table 2). Amyloid PET was available in 885 subjects (florbetaben: n=307, Florbetapir: n=390, NAV4694: n=2), and tau PET was scanned using AV-1451 tracer only and was available in 662 subjects. Age, sex, education and age differed across diagnostic groups, therefore included as covariates in downstream ADNI analyses. As expected, cognitive performance declined across diagnostic stage, with lower MoCA and MMSE scores in MCI and AD compared with cognitively normal participants (MoCA: t = −14.96, P < 0.0001; MMSE: t = −16.70, P < 0.0001). AD molecular biomarkers showed the expected diagnostic gradient, with progressively higher global amyloid SUVR, Early Amyloid Composite SUVR, global tau SUVR and meta-temporal tau SUVR from cognitively normal participants to MCI and AD (amyloid: t = 10.47, P < 0.0001; tau: t = 8.05, P < 0.0001).

### 3.2 Validation of the proposed models

We first evaluated the performance and robustness of the regional brain age prediction models. The GM regional brain age model was based on our previously validated cortical GCN framework; therefore, the present validation focused primarily on the newly developed FLAIR-derived WM regional brain age models.

Across held-out healthy UK Biobank test folds, the WM regional models showed consistently low prediction error (Table 3). Mean absolute error (MAE) ranged from 1.76 to 2.47 years across lobar and depth-specific WM regions. Mean prediction error was similar between hemispheres, with average MAE of 2.17 years for the left hemisphere and 2.20 years for the right hemisphere. Prediction performance was broadly stable across WM depth compartments and lobar regions, supporting the reliability of the multi-depth FLAIR-based surface representation for regional WM brain age estimation.

We next assessed whether WM BAG captured information related to established WM pathology while remaining distinct from conventional lesion burden. Global WM-BAG was significantly associated with WMH burden, but the effect size was small (Pearson’s r = 0.079, p < 0.0001; Supplementary Fig. 1). This weak association indicates that WM-BAG is not simply a surrogate for WMH volume and may capture additional variation in normal-appearing or diffusely altered WM that is not summarized by lesion burden alone.

We further evaluated possible age-related and acquisition-related bias. Bland–Altman analysis showed no clear residual dependence of corrected WM-BAG on chronological age (β = −0.019, t = −1.89, P = 0.1; Supplementary Fig. 2), supporting the effectiveness of age-bias correction. WM-BAG distributions were also comparable across the three UK Biobank imaging sites (Supplementary Fig. 2–3). Covariate-adjusted site effects were non-significant (site effect: F = 0.044, P = 0.957; Supplementary Fig. 3), suggesting that WM brain age estimates were not dominated by site-specific acquisition effects.

Finally, we compared regional BAG with their constituent imaging features to test whether machine-learning-derived measures provided information beyond conventional structural summaries. For GM, regional GM-BAG showed stronger or comparable associations with clinical and cognitive variables relative to cortical thickness and GM/WM intensity contrast (Supplementary Tables 1–2). For WM, WM-BAG generally showed stronger associations than WMH burden across several vascular, metabolic and cognitive variables, including composite metabolic syndrome score, alcohol use, cholesterol, BMI, HbA1c, hypertension and cognition. The strongest feature-comparison effect was observed for BMI (BAG: β = 0.029, P < 0.0001; constituent feature: β = 0.083, P = 0.069). These comparisons support the use of regional BAG as a summary marker that captures clinically relevant variation beyond individual MRI-derived features alone.

### Regional brain age associations with cardiometabolic and lifestyle risk

We next tested whether regional GM and WM BAGs were associated with cardiometabolic and lifestyle risk factors in UK Biobank. Cardiometabolic burden derived from hypertension, obesity, hypercholesterolaemia and diabetes-related measures, was summarized using a composite metabolic syndrome score (CMS). Each component was categorized according to established clinical thresholds from current guidelines^26–29^. Higher CMS values indicate greater cardiovascular risk burden. Subjects with missing values in any component were excluded from CMS calculation. We also examined individual alcohol consumption and smoking as lifestyle variables.

As shown in Fig. 2, our analysis revealed a clear dissociation between WM and GM in their relationships with cardiovascular risk factors. WM-BAGs showed widespread associations with CMS, alcohol, and smoking, whereas GM-BAGs showed no association but with alcohol.

Notably, the association of WM-BAGs with CMS and lifestyle factors were markedly more pronounced in individuals younger than 65 years, which was also significant in the analysis of interaction term of CMS ^x^ age, highlighting a stage-dependent effect in which vascular influences on brain aging are more pronounced in midlife. This pattern is consistent in the analysis of association between single risk factors and regional BAGs (Supplementary Fig. 4).

### BAG correlation with cognitive scores

We then examined associations between regional BAGs and cognitive performance. Across cognitive domains, higher BAG was generally associated with lower cognitive performance, indicating that older-appearing regional brain structure was linked to poorer cognition (Fig. 3).

The spatial pattern of these associations differed between GM and WM. WM-BAG showed stronger associations with cognition in participants aged ≥65 years, with negative associations observed across multiple WM compartments (strongest WM association: region = temporal pvWM, β = −0.31, t = −4.01, P_FDR < 0.0001). pvWM WM-BAG most strongly correlated with cognition than other depth regions, supported by a significant depth-by-cognition interaction (interaction: depth × cognition, t = −2.79, P = 0.0053).

In contrast, GM-BAG showed widespread negative associations with cognition that were more pronounced in participants aged <65 years (strongest GM association: region/network = Salient, β = −0.76, t = −3.77, P_FDR = 0.0015). These associations were observed across cognitive domains, including executive function and processing speed (executive function: network = DMN, β = −0.46, P_FDR = 0.018; processing speed: network = auditory, β = −0.55, P_FDR < 0.0001; Supplementary Fig. 5).

### Mediation analysis

To further investigate the mechanistic link between cardiovascular risk, brain aging, and cognition, we performed mediation analyses using compartment-level WM brain age measures. First, the original 12 region-specific WM-BAGs were averaged to represent global WM-BAG and averaged within each depth of pvWM, dWM, and jxWM to improve interpretability at the systems level.

Significant mediation effects were observed in participants aged <65 years (Fig. 4). In this age group, global WM-BAG statistically mediated the association between CMS and cognitive performance (indirect effect a × b = −0.023, 95% CI [−0.0054 −0.00010], P =0.039). pvWM-BAG also showed a significant indirect effect (a × b = −0.0029, 95% CI [−0.0059 −0.00042], P = 0.021), indicating that periventricular WM brain aging accounted for part of the CMS–cognition association. The indirect effect was numerically larger for global WM-BAG than for pvWM-BAG (global WM: a × b = −0.023; pvWM: a × b = −0.0029), whereas pvWM provided a more anatomically localized mediator.

### ADNI group analysis

We next examined whether regional BAGs differed across diagnostic groups in ADNI. Regional BAGs differed across diagnostic groups (Fig. 5). The CN versus MCI contrast showed modest but significant differences in frontal juxtacortical WM and auditory GM (WM: t = 7.54, P_FDR < 0.0001; GM: t = 6.38, P_FDR < 0.0001).In contrast, the MCI versus AD contrast showed stronger effects, with higher BAG in AD across both WM and GM measures (WM: t = 5.26, P_FDR < 0.0001; GM: t = 9.80, P_FDR < 0.0001).

The strongest diagnostic-stage effects were observed in frontal WM and default mode network GM regions. AD participants showed higher frontal WM-BAG than MCI participants (β = 2.09, t = 5.21, P_FDR < 0.0001) and higher default mode GM-BAG than MCI participants (β = 3.64, t = 9.80, P_FDR < 0.0001). These effects were larger than those observed in the CN versus MCI comparison (CN versus MCI, frontal WM: β = 1.65, t = 7.49, P_FDR < 0.0001; CN versus MCI, default mode GM: β = 1.53, t = 4.92, P_FDR < 0.0001).

### Correlation between SUVR uptakes and regional BAGs

In analyses restricted to cognitively normal and MCI participants, PET-related BAG associations were modest and spatially limited (Fig. 6). Amyloid burden showed positive associations with BAG in frontal jxWM and auditory, default mode and limbic networks, (amyloid–frontal jxWM-BAG: β = 2.04, t = 3.17, P_FDR = 0.019; amyloid–auditory GM-BAG: β = 2.62, t = 3.67, P_FDR = 0.0024; amyloid–default mode GM-BAG: β = 1.67, t = 2.94, P_FDR = 0.015; amyloid–limbic GM-BAG: β = 1.70, t = 2.76, P_FDR = 0.018). Tau-related associations were more evident for WM-BAG than amyloid-related associations, particularly in frontal WM compartments ((tau–frontal jxWM-BAG: β = 3.80, t = 6.94, P_FDR < 0.0001; tau–frontal dWM-BAG: β = 2.93, t = 5.85, P_FDR < 0.0001; tau–frontal pvWM-BAG: β = 2.70, t = 5.36, P_FDR < 0.0001). Amyloid PET sensitivity analyses stratified by tracers showed broadly consistent directions of association for florbetaben and florbetapir, with no significant interaction effects between the two tracers’ SUVR and the BAGs in any subregions.

When analyses were extended to all participants, including CN, MCI and AD groups, PET-related associations became stronger and more spatially widespread. Tau burden showed broad positive associations with WM-BAG, with strongest effects observed in the 3 frontal WM depths (strongest tau–WM association: frontal jxWM, β = 4.21, t = 8.96, P_FDR < 0.0001). Amyloid burden also showed positive associations with regional BAG, with effects observed in all WM compartments as well as broadly across GM networks (strongest amyloid–WM association: frontal jxWM, β = 3.08, t = 5.25, P_FDR < 0.0001). GM-BAG associations with amyloid PET SUVR were most evident in auditory network (β = 4.53, t = 6.99, P_FDR < 0.0001).

## Discussion

In this study, we evaluated whether machine-learning-derived brain age can be decomposed into tissue- and compartment-specific signatures that map the distinctive trajectories of the human brain aging. Across two large cohorts, T2-FLAIR-derived WM brain age and T1-derived GM brain age captured partially separable biological and clinical associations. Cardiovascular and metabolic risk burden was preferentially associated with accelerated WM brain aging, particularly in adults younger than 65 years, whereas GM brain age showed more limited associations. WM brain age, predominantly within the periventricular WM, mediated the association between cardiometabolic burden and cognition in midlife. Across the AD continuum, regional GM and WM brain age both increased with diagnostic severity but showed different pathological associations: GM brain age aligned more closely with amyloid-related disease burden, whereas WM brain age showed prominent associations with tau burden. These findings suggest that the major value of tissue-resolved brain age lies not in producing additional regional predictions alone, but in revealing complementary vascular-metabolic and neurodegenerative signatures that are obscured by global brain age estimates. Therefore, this framework moves brain age beyond a single aggregate index toward a scalable set of imaging phenotypes that better reflect the heterogeneity of human brain aging.

A major finding was the preferential association between cardiometabolic risk and WM brain age in midlife. Cardiovascular and metabolic risk factors are well-established drivers of systemic microvascular dysfunction, which manifests within the central nervous system as cerebral small vessel disease, WM injury, and subsequent cognitive decline^17,30^. MH is a core imaging manifestation of small vessel disease, but represents the overt end of a broader spectrum of WM abnormalities^31^. Recent studies of *dirty-appearing* or DAWM provide a related perspective: subtle FLAIR abnormalities outside conventional WMH have been associated with subsequent progression of small vessel disease, including increasing WMH burden and incident subcortical infarcts^12^. At the same time, recent longitudinal evidence suggests that DAWM alone may not predict subsequent cognitive decline or dementia, underscoring that subtle FLAIR abnormalities are biologically heterogeneous and should not be equated directly with clinical outcomes^13^. In this context, our T2-FLAIR-derived WM brain age provides a complementary approach by integrating unthresholded signal variation across the WM rather than defining a visually delineated DAWM compartment or relying on WMH volume alone. The weak association between WM BAG and WMH burden suggests that the model captures additional distributed variation in WM signal. Although FLAIR signal is biologically nonspecific, such variation may reflect the cumulative effects of vascular injury, microstructural tissue alteration and altered tissue water content that accompany WM aging. Indeed, our framework demonstrates that a composite cardiovascular and metabolic risk score during midlife was preferentially associated with WM brain age, whereas its association with GM was relatively limited. This finding in the midlife group highlights the emerging view that subtle WM abnormalities may precede or accompany overt WMH in cerebral small vessel disease, which could be potentially driven by cumulative cardiovascular and metabolic burdens. This is important because midlife vascular risk has been linked to later-life brain structure, WMH burden and cognition, including evidence that vascular contributions to cognitive decline may operate partly independent of amyloid pathology^32^.

The regional organization of WM brain age further refined this vascular-metabolic pathway by identifying the compartments most strongly mediating the link between cardiometabolic burden and cognition. Our analysis reveals that pvWM brain age provides a more anatomically localized mediator^17,33^. This pattern is biologically plausible because pvWM is a canonical site of small-vessel disease-related injury and lies along long-range projection and association pathways that support distributed cognitive function^31,34,35^. Thus, depth-resolved WM brain age may help separate vascular-dominant WM vulnerability from other patterns of WM aging that are more closely coupled to cortical or juxtacortical neurodegenerative processes. This provides a stronger clinical rationale for modelling WM aging by compartment rather than treating WM as a homogeneous structure. This finding also highlights why global WM measures may be insufficient: averaging across juxtacortical, deep and periventricular compartments can obscure spatially localized aging pathways with distinct clinical relevance. However, our mediation results should not be interpreted as proof of causal mediation, because the data are observational and largely cross-sectional.

Our tissue-specific framework further extends into neurodegenerative processes across the AD continuum. In the ADNI cohort, GM and WM brain age both increased across diagnostic stages, but they showed different relationships with molecular pathology. GM brain age broadly correlated with neocortical amyloid burden, whereas WM brain age showed a distinct association with tau accumulation within the temporal meta-ROI during the transition from CN to MCI. Importantly, WM brain age should not be interpreted as a surrogate marker of tau pathology. Rather, because T2-FLAIR-derived WM brain age likely reflects a heterogeneous combination of vascular injury, blood-brain barrier dysfunction, demyelination, axonal degeneration, gliosis and tissue water alterations^36–39^. This interpretation is consistent with emerging evidence that vascular-related WM injury and tau pathology progress in parallel during early AD and may exacerbate one another. Cerebrovascular dysfunction can impair blood-brain barrier integrity and axonal transport, thereby facilitating tau accumulation and propagation, while tau-related neurodegeneration can further disrupt WM integrity. Previous studies have similarly shown that WM degeneration is closely linked to tau pathology and may emerge alongside, or even precede, overt cortical degeneration^40,41^. Recent diffusion MRI studies further highlight the importance of WM integrity for understanding how AD pathology translates into cognitive dysfunction^42^. Together, these findings suggest that T2-FLAIR-derived WM brain age captures a complementary vascular-microstructural pathway that intersects with tau-related neurodegeneration, rather than serving as a direct proxy for tau burden.

A key contribution of this work is conceptual and methodological. Most brain age models derive a single global estimate, often from T1-weighted structural MRI or cortical and whole-brain morphology^43–45^. Recent studies have extended this paradigm using multimodal neuroimaging or anatomically interpretable regional models, starting tests of whether different tissue compartments capture distinct biological dimensions of aging^46–48^. Furthermore, our framework jointly models GM and WM brain age within a unified surface-based architecture, integrating T1-weighted and T2-FLAIR MRI to generate region-specific aging estimates across anatomically and functionally organized tissue systems. The predefined regional organization was chosen to balance biological interpretability with model scalability. GM regions were defined using large-scale functional networks because these systems provide established units for studying cognition and neurodegeneration. WM regions were defined using lobar anatomy and cortical-to-ventricular depth because pvWM, dWM and jxWM differ in vascular supply, connectivity architecture, lesion susceptibility and proximity to cortical association systems^49,50^. This organization enabled us to test biologically motivated hypotheses regarding vascular-metabolic burden, cognition and Alzheimer’s disease pathology. This design also addresses a broader issue in brain age research. Brain aging can be represented at multiple spatial scales, from global brain age estimates to tissue-specific regional models and, eventually, spatially continuous maps of aging vulnerability. Global models provide useful summary measures but may collapse biologically distinct processes into a single index. Fully data-driven spatial decompositions may reveal latent patterns but can be difficult to interpret clinically or compare across cohorts. The present framework occupies an intermediate scale: it preserves anatomical and functional organization while resolving brain age into interpretable tissue and compartment signatures. The distinct associations observed for GM and WM brain age across cardiometabolic risk, cognition and AD-related pathology suggest that machine-learning-derived brain age captures integrated patterns of structural variation that are not readily summarized by conventional imaging measures alone.

Despite these findings, several limitations warrant consideration. First, the cross-sectional design of both the UK Biobank and ADNI cohorts precludes establishing definitive temporal or causal relationships between cardiometabolic risk, brain aging and cognitive decline. Longitudinal studies will be required to determine whether the observed WM and GM aging signatures predict future cognitive and clinical outcomes. Second, both cohorts exhibit limited racial and ethnic diversity, and future work is needed to establish the generalizability of this framework across more diverse populations. Third, T2-FLAIR signal is biologically nonspecific and may reflect multiple tissue and acquisition-related factors, including demyelination, gliosis, axonal injury, interstitial water content, partial-volume effects and scanner-dependent intensity variation. Fourth, the regional WM representation based on juxtacortical, deep and periventricular compartments provides a tractable and interpretable framework, but does not map directly onto specific vascular territories, fiber populations or pathological mechanisms. Finally, brain age is a predictive imaging construct rather than a direct measure of biological aging. Future studies integrating longitudinal imaging, diffusion MRI, myelin-sensitive imaging, histopathological validation and more representative population cohorts will be important for refining the biological interpretation and clinical utility of tissue-specific brain age measures.

In conclusion, developing a unified framework capturing continuous T1-derived GM and T2-FLAIR-derived WM brain age metrics reframes brain aging as a set of complementary tissue- and compartment-resolved imaging phenotypes. This dual-tissue framework identifies preferential associations between WM aging and midlife vascular-metabolic burden, pvWM aging and cognition, and distinct GM and WM associations with amyloid and tau pathology across the Alzheimer’s disease continuum. More broadly, the study positions brain age methods along a continuum from global summary indices toward interpretable regional and tissue-resolved models, with future work extending toward longitudinal and spatially continuous representations of aging vulnerability.

## Methods

### Overview

We developed a multimodal, regional brain age prediction framework to estimate tissue- and region-specific brain aging from paired T1-weighted and T2-FLAIR MRI (Fig. 1). The framework comprised two parallel surface-based representations. First, a gray matter (GM) representation was generated from T1-weighted MRI by reconstructing cortical surfaces, extracting vertex-wise cortical features and parcellating the cortex into functional regions, which follows the method described in our prior work^25^. Second, a white matter (WM) representation was generated by defining a continuous WM domain between the GM–WM interface and the lateral ventricles, constructing a Laplacian depth field, extracting intermediate WM surfaces and sampling T2-FLAIR signal across lobar and depth-specific WM compartments.

These representations were used as inputs to regional graph convolutional network (GCN)-based brain age prediction models. The GM models estimated regional cortical brain age from T1-derived cortical thickness and GM/WM intensity contrast. The WM models estimated regional WM brain age from FLAIR signal sampled on multi-depth WM surfaces. Regional BAG was then calculated as predicted brain age minus chronological age and used to test tissue- and region-specific associations with vascular and metabolic risk, cognition and AD-related biomarkers.

### Participants and datasets

Participants were included if they had paired T1-weighted and T2-FLAIR MRI scans that passed image-processing and quality-control procedures. Participants were excluded if they had incomplete imaging, failed cortical surface reconstruction, inaccurate T2-FLAIR-to-T1 registration, poor WM or ventricular segmentation, or major structural abnormalities that could substantially distort brain age estimation, including large territorial infarcts, brain tumors, major surgical defects or severe imaging artefacts.

The primary dataset was the UK Biobank, a large population-based cohort designed to investigate the determinants of health and disease in middle-aged and older adults^20^. Participants with documented major neurological disease were excluded where available; vascular and metabolic conditions were retained for downstream association analyses rather than excluded. The UK Biobank analysis initially included 27,234 and finally consisted of 26,446 participants without neurological disease and with paired T1-weighted and T2-FLAIR MRI scans that passed image-processing and quality-control procedures (Table 1, 64.7 ± 7.5 years, with 50.76% male). The base brain age prediction model was trained using subjects from UK Biobank, excluding those with any documented diseases and vascular and metabolic conditions.

To evaluate generalizability and relevance in relation to neurodegeneration, we further included participants from the Alzheimer’s Disease Neuroimaging Initiative (ADNI), a longitudinal, multi-site study of AD and related cognitive decline^21^. We used ADNI phase 2-4 cohorts and the ADNI sample initially included 1,006 and finally comprised 992participants with paired T1-weighted and T2-FLAIR MRI scans that passed image-processing and quality-control procedures, including 480 cognitively normal individuals, 328 participants with mild cognitive impairment and 184 participants with AD. We fine-tuned the brain age prediction model from UK BioBank using ADNI cognitively normal data from cognitively unimpaired participants

All participants provided written informed consent under the protocols of the respective parent studies.

### MRI acquisition and preprocessing

MRI data from the UK Biobank were acquired on Siemens Skyra 3T scanners using harmonized imaging protocols across sites. T1-weighted images were obtained using a 3D MPRAGE sequence (repetition time TR ≈ 2000 ms, echo time TE ≈ 2–3 ms, inversion time TI ≈ 800–900 ms, voxel size ≈ 1 × 1 × 1 mm^3^). FLAIR images were acquired using a 3D T2-FLAIR sequence (TR ≈ 5000 ms, TE ≈ 300–400 ms, TI ≈ 1800 ms, voxel size ≈ 1 × 1 × 1 mm^3^), optimized for WM lesion detection.

ADNI MRI data were acquired across multiple sites on Siemens and GE scanner platforms following standardized acquisition protocols for 3-Tesla MRI. T1-weighted images were acquired using 3D MPRAGE sequences (TR ≈ 2300 ms, TE ≈ 2–4 ms, TI ≈ 900 ms, voxel size ≈ 1 × 1 × 1.2 mm^3^), while FLAIR images were acquired using T2-FLAIR sequences (TR ≈ 9000 ms, TE ≈ 90–140 ms, TI ≈ 2500 ms, voxel size ≈ 1 × 1 × 1.2 mm^3^), with minor variations across sites.

All images underwent standard preprocessing, including skull stripping and intensity normalization. T1-weighted MRI images were then linearly registered to the MNI152 template^51^, and T2-FLAIR images were subsequently aligned to the corresponding T1 images in the MNI template space. This ensures spatial consistency across modalities and subjects, enabling accurate surface representation and reliable sampling of T2-FLAIR signals at anatomically corresponding locations. Surface reconstruction and segmentation were performed using FreeSurfer version 6.0, following established pipelines.

All preprocessing outputs were visually and/or automatically quality controlled. Bias-field correction, brain extraction, image registration and surface alignment were visually inspected by trained personnel. Surface reconstruction quality was further assessed using automated scripts that evaluated anatomical validity and mesh integrity, including whether vertices were located within the expected tissue compartments and whether surfaces contained holes, discontinuities, self-intersections or abnormal mesh intersections. Participants with failed T1-weighted surface reconstruction, inaccurate T2-FLAIR-to-T1 registration, poor ventricular or white matter segmentation, defective white matter surface reconstruction, or incomplete paired T1-weighted and T2-FLAIR data were excluded from downstream analyses.

### GM regional and multi-depth WM surface representation

#### GM cortical surface representation

T1-weighted MRI scans were processed using FreeSurfer v6^52^ with recon-all pipeline to reconstruct cortical surfaces and generate vertex-wise cortical measures. The GM–WM and pial surfaces were reconstructed for each hemisphere, and cortical thickness and GM/WM intensity contrast were extracted at each cortical vertex^25^. Surface features were resampled to a common surface space to enable vertex-wise correspondence across participants.

The cortical surface was parcellated into nine functional regions derived from the atlas defined in Yeo et al^53^. These regions comprised sensorimotor, frontoparietal, dorsal attention, ventral attention/language, default mode, salience, auditory, visual and limbic networks as also defined in our previous studies^25,54^. Homologous left- and right-hemisphere regions were merged for GM regional brain age prediction. For each GM region, the corresponding cortical vertices and mesh adjacency relationships were used to construct a regional graph, with cortical thickness and GM/WM intensity contrast serving as node-wise input features.

#### Multi-depth WM surface representation

To characterize the T2-FLAIR-derived WM signal across tissue depths, we constructed a subject-specific multi-depth WM surface representation between the GM–WM interface and the lateral ventricles. The outer boundary of the WM domain was defined by the FreeSurfer-derived GM–WM surface, and the inner boundary was defined by the lateral ventricular segmentation. The T2-FLAIR image was registered to the corresponding T1-weighted image before WM surface sampling, and FLAIR intensity was normalized within each participant using whole-WM mean and standard deviationto reduce inter-individual intensity-scaling differences.

Within this WM domain, we solved a Laplacian depth field to define a continuous normalized coordinate between the GM–WM interface and the ventricular boundary. Ten isosurfaces were generated from this field, spanning the cortical-to-ventricular WM axis. The two boundary surfaces corresponding to the GM–WM interface and ventricular surface were excluded, leaving eight internal WM surfaces for analysis. These surfaces were grouped into three anatomically motivated depth compartments: the two outermost surfaces closest to cortex were assigned to juxtacortical WM (jxWM), the three intermediate surfaces to deep WM (dWM), and the three innermost surfaces closest to the ventricles to periventricular WM (pvWM). The use of two surfaces for jxWM was chosen to reflect the relatively narrow and folded superficial WM zone immediately beneath the cortex, which contains short association/U-fibers unlike deeper WM compartments^50,55,56^.

Each WM surface was further parcellated into four lobar regions: frontal, parietal, temporal and occipital. Lobar labels were defined on the GM–WM surface and propagated to the intermediate WM surfaces, yielding WM regions defined by hemisphere, lobe and depth compartment. To establish vertex-wise correspondence across participants, surface registration was performed separately at each WM depth. One participant was randomly selected as the initial reference template, and surfaces from 400 randomly selected participants were registered to this template using mris_register. The registered surfaces were then averaged using mris_make_template to generate a population-based template for that depth. All participants were subsequently registered to the corresponding depth-specific template. This template construction and registration procedure was repeated iteratively until the vertex coordinates showed no appreciable change between successive iterations. Each hemispheric WM surface was then resampled to a level-6 icosahedral mesh containing 40,962 vertices, matching the surface resolution used for GM processing. Normalized T2-FLAIR signal was interpolated at corresponding vertices using cubic B-spline interpolation, producing regional WM feature matrices for downstream graph convolutional brain age prediction. Additional technical details are provided in Method S1.

#### Regional brain age prediction models

We developed a unified deep learning framework for regional brain age prediction to estimate tissue- and region-specific brain age from surface-based MRI features. Both GM and WM models used GCNs, which learn from imaging features defined on the aforementioned surfaces that represent vertices as graph nodes and mesh adjacency relationships as graph edges. This framework extends our previous regional cortical brain age prediction approach^25^. In spectral GCNs, node-wise imaging signals are filtered with respect to the graph Laplacian, enabling convolution-like feature learning on non-Euclidean surface geometry.

For GM brain age prediction, we used T1-weighted MRI-derived cortical thickness and GM/WM intensity contrast sampled at each cortical vertex. Regional GM graphs were defined within the previously described nine functional cortical subregions, with homologous left- and right-hemisphere regions combined.

For WM brain age prediction, we used normalized T2-FLAIR signal sampled from the multi-depth WM surface representation described above. Each WM surface parcellated into 4 lobes was combined with homologous left- and right-hemisphere regions, yielding regional WM compartments defined by lobe and depth. For each WM region, T2-FLAIR signals sampled at corresponding vertices across the surfaces within a given depth compartment were used as node-wise input channels for the regional GCN; jxWM models used two channels, whereas dWM and pvWM models used three channels.

All models were trained to predict chronological age in the UK Biobank training sample using five-fold cross-validation. Within each fold, imaging features were standardized using parameters estimated from the training set and then applied to validation and test data to avoid information leakage. Models were trained using Kullback–Leibler divergence loss between predicted and target age distributions and optimized using Adam. The fivefold-specific models were ensembled by averaging their predicted ages. Regional BAG was calculated as predicted regional brain age minus chronological age. To mitigate age-related bias in predicted age difference, we applied the linear trend removal method proposed by Smith and colleagues before statistical analyses. More technical details are provided in Method S2.

### Statistical analysis

Statistical analyses were performed separately for GM and WM regional BAG unless otherwise specified. Regional brain age model performance was evaluated in held-out UK Biobank folds using mean absolute error, root mean squared error and the correlation between predicted and chronological age. Age-related bias in predicted age difference was assessed before and after correction using the linear trend removal method proposed by Smith and colleagues. Model calibration was visualized using Bland–Altman plots, and residual associations between corrected BAG and chronological age were quantified. Robustness to acquisition-related variability was assessed by examining regional BAG distributions across imaging sites and by estimating covariate-adjusted site effects.

We tested associations between regional BAG and cardiovascular/metabolic risk factors, cognitive performance and Alzheimer’s disease-related biomarkers using linear or logistic regression models, as appropriate. Models included covariates for chronological age, sex, imaging site/scanner and other analysis-specific variables. For analyses involving clinical measures acquired at a different time from MRI, the interval between MRI and clinical assessment was included as an additional covariate. Analyses were performed in the full sample and stratified into midlife adults aged <65 years and older adults aged ≥65 years to test whether tissue-specific associations differed across adulthood. Multiple comparisons across regions and outcomes were controlled using false discovery rate (FDR) correction.

To determine whether machine-learning-derived brain age provided information beyond conventional imaging features, we compared regional BAGs with their constituent MRI features. For GM regions, these features included cortical thickness and GM/WM intensity contrast. For WM regions, they included regional FLAIR signal summaries from the corresponding depth and lobar compartments. We used nested regression models to test whether adding regional BAG improved the explanation of cardiovascular, cognitive or molecular outcomes beyond conventional structural measures alone.

In the UK Biobank cohort, we performed mediation analyses to test whether regional GM or WM BAG statistically mediated associations between cardiovascular/metabolic risk burden and cognitive performance. Mediation models estimated direct, indirect and total effects, with confidence intervals obtained using non-parametric bootstrapping. Because the analyses were observational, mediation results were interpreted as evidence of indirect statistical association rather than causal mediation.

In ADNI, we examined disease-stage differences in regional BAG across cognitively normal, mild cognitive impairment and Alzheimer’s disease groups. Planned contrasts compared cognitively normal versus mild cognitive impairment and mild cognitive impairment versus Alzheimer’s disease. We further examined associations between regional BAGs and PET biomarkers of Alzheimer’s disease pathology, including amyloid-β and tau SUVRs. Primary PET analyses were conducted in the full ADNI PET sample, with secondary analyses focused on cognitively normal and mild cognitive impairment participants to assess early disease-stage associations.

Because amyloid PET data in ADNI were acquired using multiple tracers, we performed sensitivity analyses to assess tracer-related robustness. Primary amyloid PET models included tracer type as a covariate. We further repeated amyloid analyses separately for florbetaben and florbetapir participants, excluding NAV4694 because of the very small sample size. Tau PET analyses used AV-1451 only. To determine whether stronger associations in the full ADNI sample were driven by diagnostic-stage composition rather than sample size alone, PET-BAG associations were additionally examined separately in CN+MCI participants, AD participants and the full CN+MCI+AD sample.

## Figures and Tables

**Figure 1:**
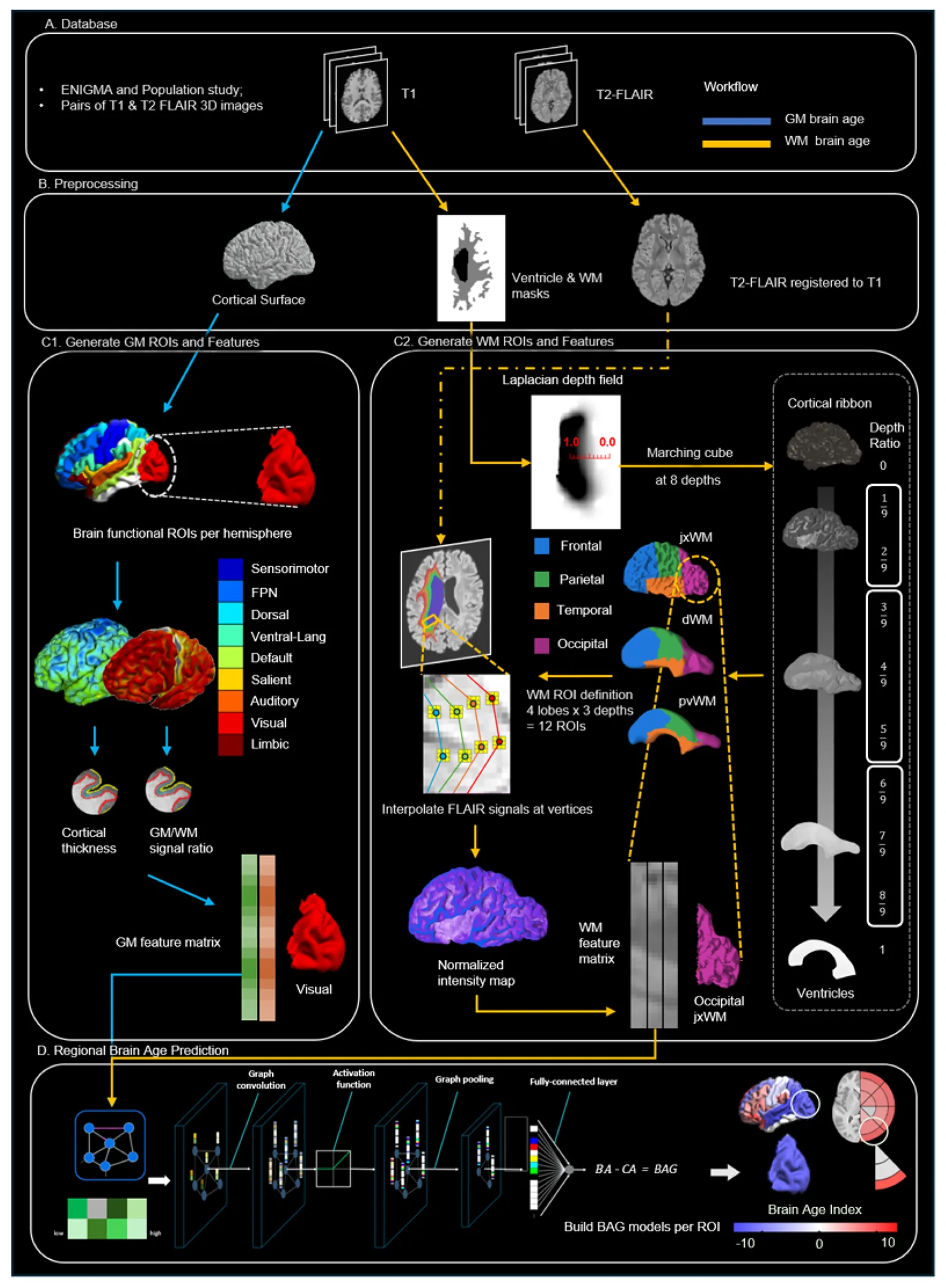
Multimodal regional brain age prediction framework for gray and white matter aging. **A.** Paired T1-weighted and T2-FLAIR MRI scans were used to construct complementary gray matter (GM; blue workflow) and white matter (WM; yellow workflow) brain age models. **B.** Preprocessing generated cortical surfaces from T1-weighted MRI, ventricular and WM masks from anatomical segmentation, and T2-FLAIR images registered to each participant’s T1-weighted image. **C1.**GM regional features were extracted from cortical surfaces parcellated into functional networks, including sensorimotor, frontoparietal, dorsal attention, ventral attention/language, default mode, salience, auditory, visual and limbic regions defined by Yeo el al. Vertex-wise cortical thickness and GM/WM intensity ratio were used to generate regional GM feature matrices (e.g. visual network region). **C2.** WM regional features were generated using a Laplacian depth field spanning the GM–WM interface to the lateral ventricles. Eight internal WM surfaces were retained after excluding the cortical and ventricular boundary surfaces, and grouped into juxtacortical WM, deep WM and periventricular WM compartments. Each surface was further parcellated into frontal, parietal, temporal and occipital lobes, yielding 12 WM regions. The normalized T2-FLAIR signal was interpolated at corresponding surface vertices to generate regional WM feature matrices (e.g. occipital jxWM). D.Regional GM and WM feature matrices were entered into graph convolutional network models, in which surface vertices were represented as graph nodes and mesh adjacency as graph edges. Each regional model predicted brain age (BA), and regional brain age gap (BAG) was calculated as predicted brain age minus chronological age (BA - CA = BAG). Regional BAG maps were then used as tissue- and region-specific brain age indices for downstream analyses. **Abbreviations:**BA, brain age; BAG, brain age gap; CA, chronological age; FPN, frontoparietal network; GM, gray matter; jxWM, juxtacortical white matter; dWM, deep white matter; pvWM, periventricular white matter; WM, white matter.

**Figure 2:**
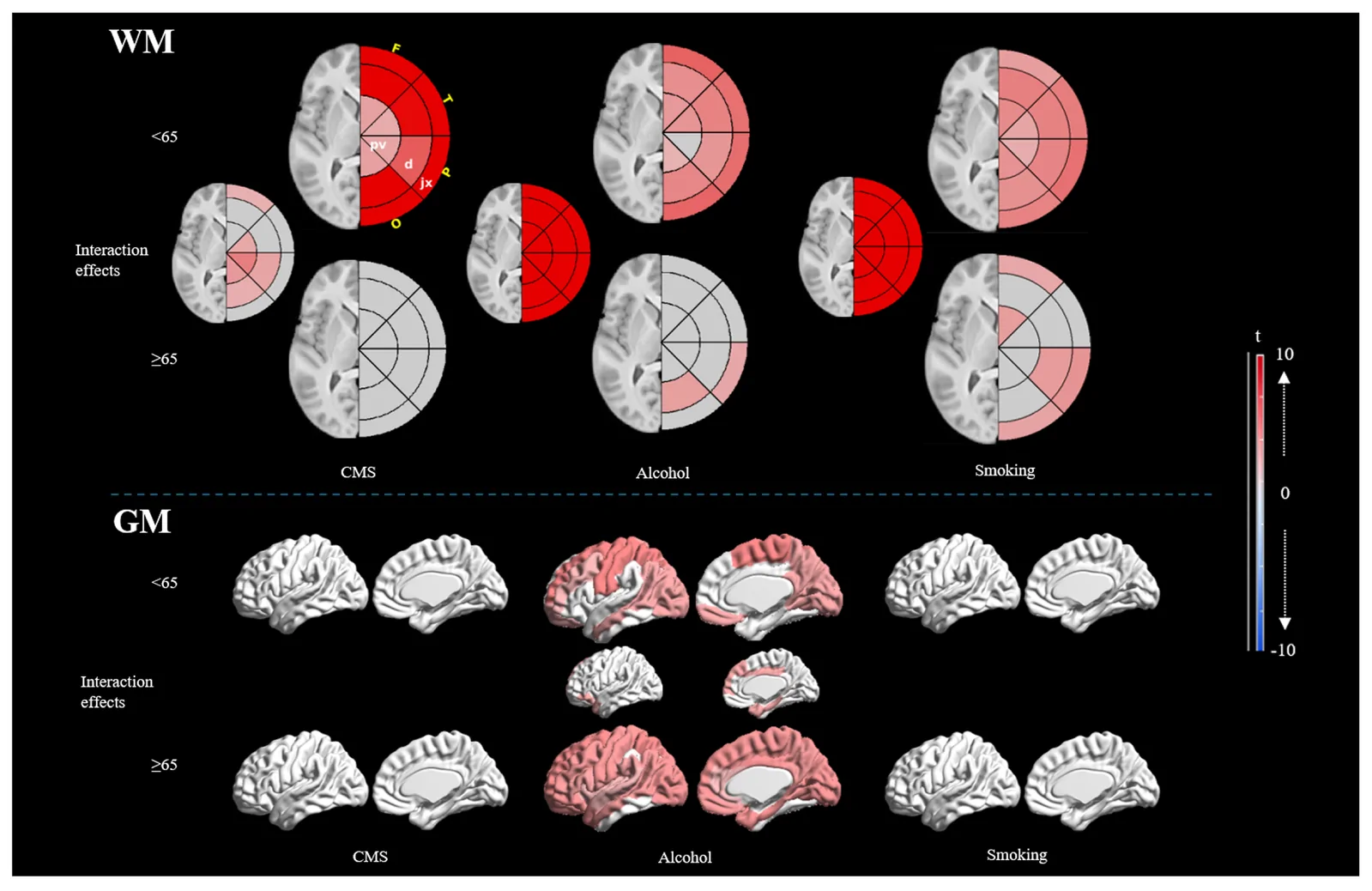
The associations between multiple cardiovascular risks and WM (top), GM (bottom) BAGs. Note that in later analysis, unless other specified, the t-value ranges from −10 to 10 and only regions with significant p-values after FDR will be shown in color.

**Figure 3:**
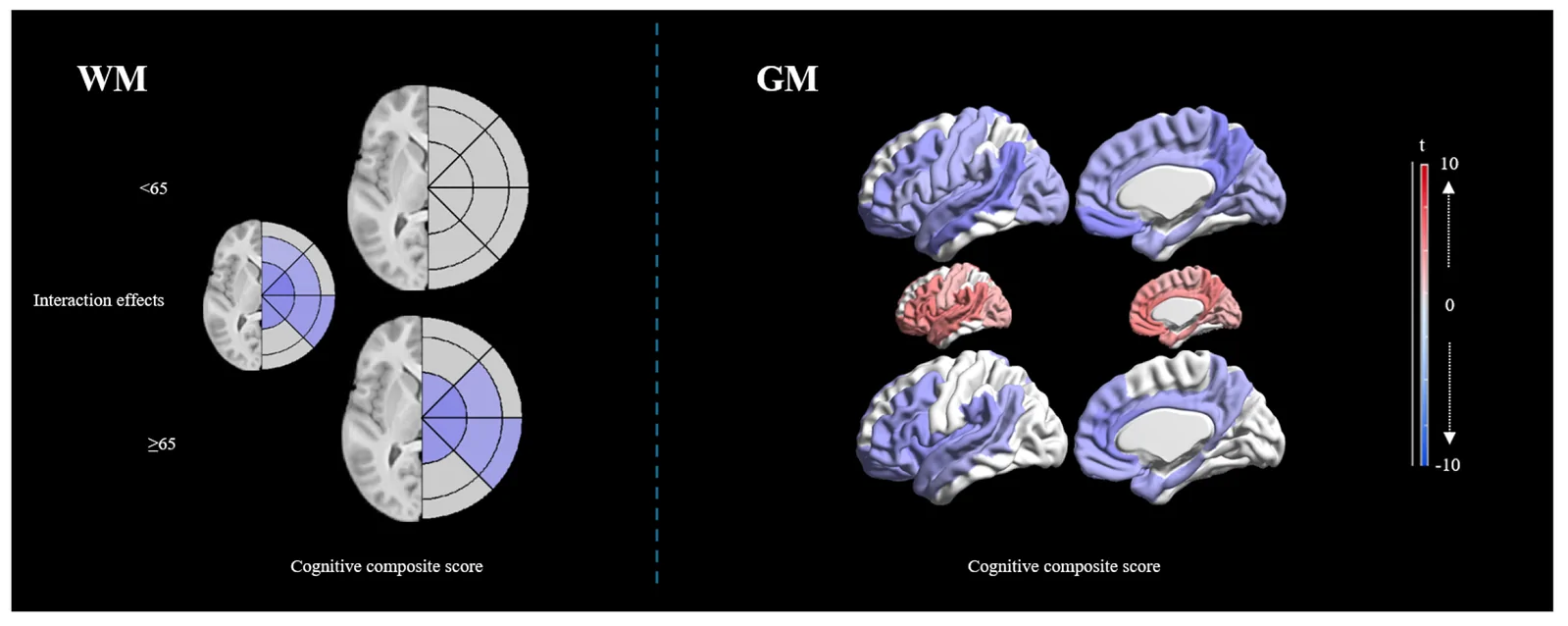
Correlations between cognitive scores and WM-BAG (left part) and GM-BAG (right part) with their interaction effects (in the middle) between group age < 65 and age ≥ 65.

**Figure 4:**
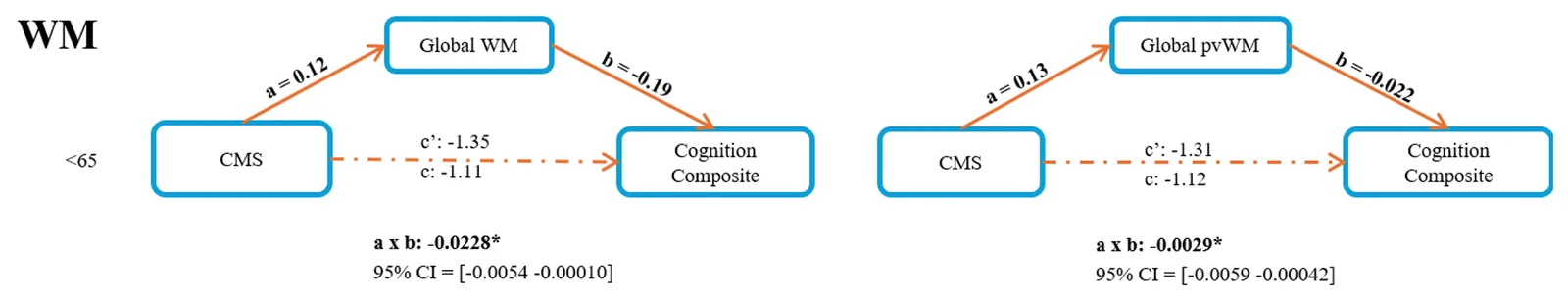
Mediation analysis results among CMS, global WM-BAG (A. left) and pvWM-BAG, and cognition composite. c: total effect; c’: direct effect; a x b: indirect effect; CI: confidence interval.

**Figure 5:**
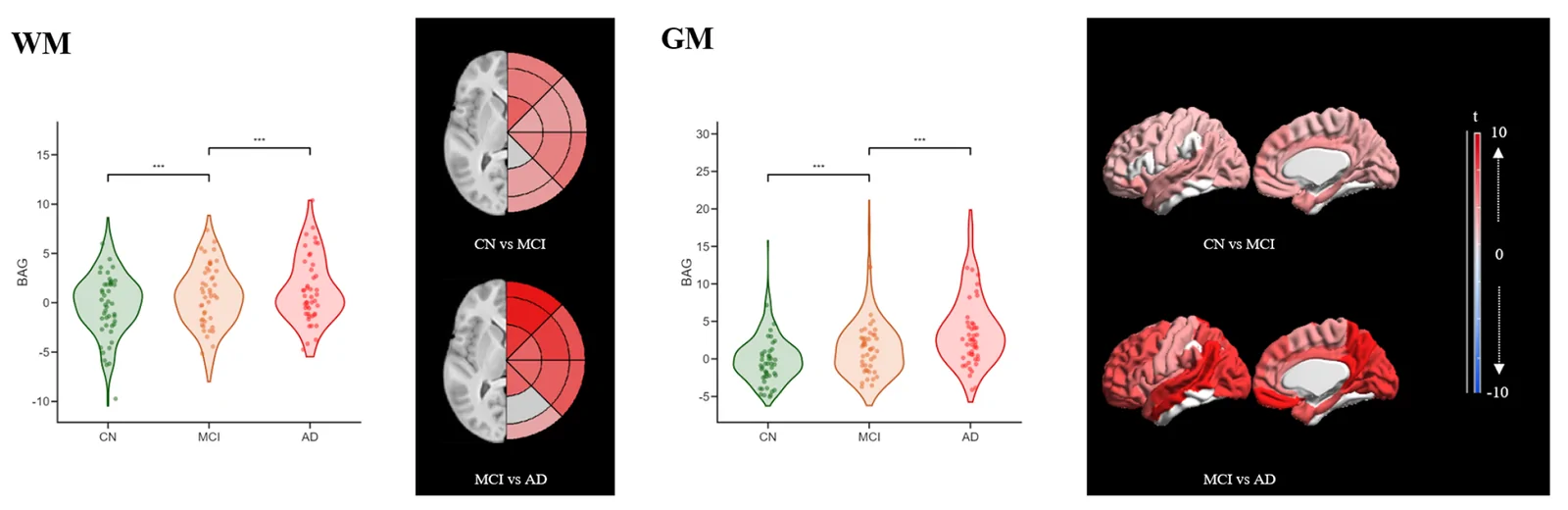
Group comparisons of the regional BAGs among CN, MCI and AD. For the 2 plots, the BAGs are averaged within all ROIs in one subject.

**Figure 6:**
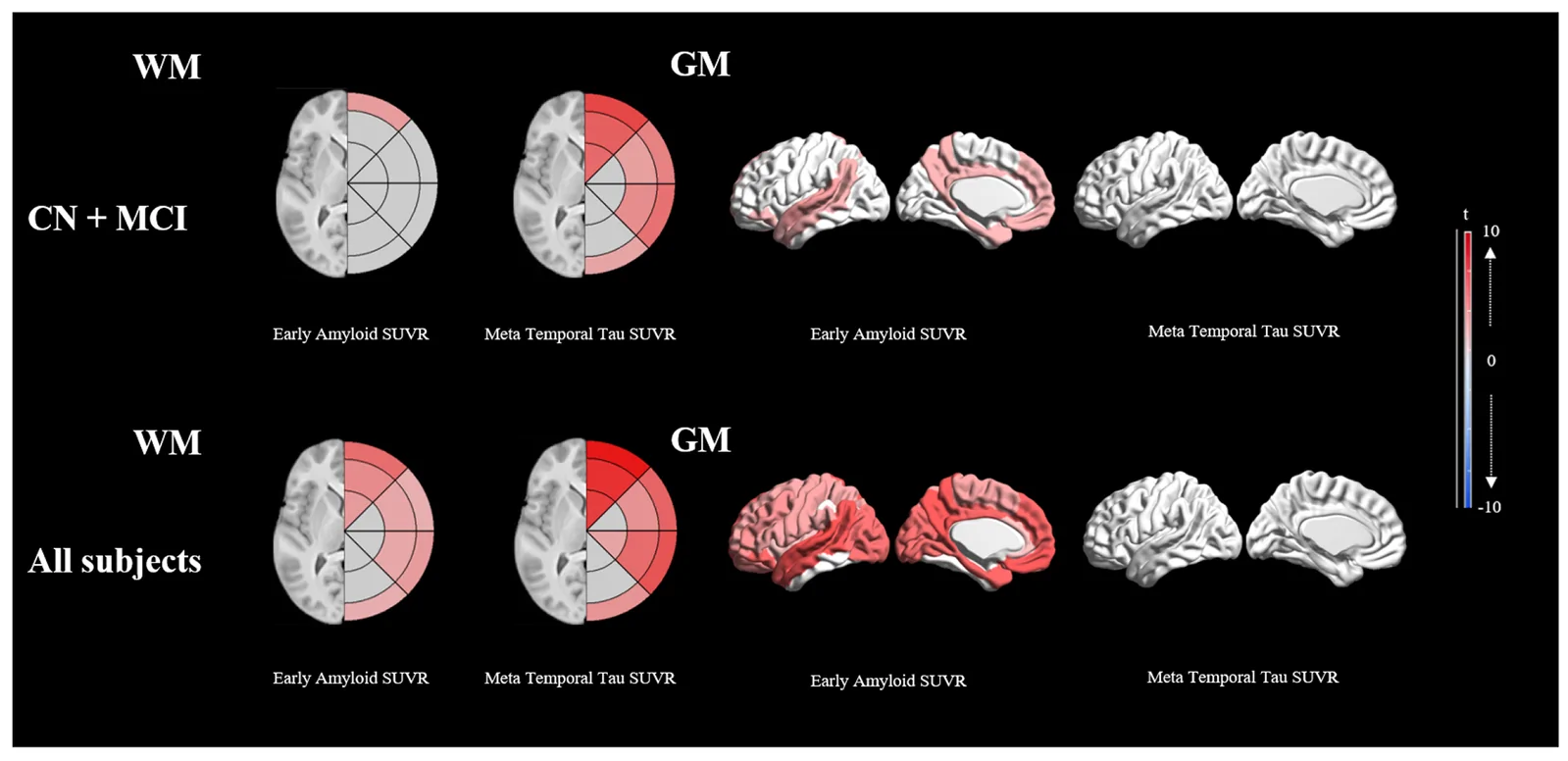
SUVR uptake differences in correlation with regional BAGs across groups. Top: CN vs MCI groups; Bottom: CN vs MCI vs AD groups; Left: WM results; Right: GM results.

**Table 1.** UKB Participants

Category	Variable	All subjects
	No. subjects (n)	26446
	Age (years)	64.7 (7.5)
Covariates	Sex -male, %	50.76
	Edu (years)	16.54 (3.89)
	Scan sites (site ID)	1 (Newcastle), 2 (Cheadle), 3 (Reading)
	Date from baseline (years)	8.84 (1.73)
Life Habits	Smoking (category)	0 (None), 1 (Ever), 2 (Current); valid n=26179
	Alcohol (frequency score)	1 (Never) to 6 (Most frequent); valid n=26256
	WMH volume (mm^3)	5384.45 (6805.75)
	BMI (kg/m^2)	27.65 (4.29)
	Obesity (n)	5995
	Diabetes (n)	2061
Cardiovascular	Hypertension (n)	6584
	Cholesterol (mg/dL)	222.38 (43.01)
	HbAlc (mmol/mol)	35.32 (5.37)
	Systolic BP (mmHg)	136.47 (17.75)
	Diastolic BP (mmHg)	77.83 (10.00)
	Pulse rate (bpm)	68.26 (11.11)
	Memory	−0.039 (0.78)
Cognitive outcomes	Executive function	−0.009 (0.77)
	Processing speed	−0.046 (0.80)
	Reasoning	−0.054 (0.83)

**Table 2.** ADNI participants

Category	Variable	CN	MCI	AD	FDR p-value
	No. subjects (n)	480	328	184	
Covariates	Age (years)	71.5 (8.0)	73.3 (7.9)	74.1 (8.0)	0.004
	Sex -male, %	35.83	52.44	54.35	<0.001
	Edu (years)	16.58 (2.51)	16.04 (2.60)	15.67 (2.55)	<0.001
	BMI (kg/m^2)	27.66 (5.76)	26.91 (5.27)	26.27 (5.28)	0.013
Cardiovascular	Systolic BP (mmHg)	132.77 (17.34)	131.24 (16.35)	133.27 (17.45)	0.173
	Diastolic BP (mmHg)	75.56 (9.55)	74.77 (9.89)	74.43 (9.47)	0.243
	Pulse rate (bpm)	66.90 (11.28)	65.40 (10.68)	65.59 (11.02)	0.570
Cognitive outcomes	MoCA	25.72 (3.03)	22.51 (4.62)	16.04 (4.56)	<0.001
	MMSE	28.98 (1.34)	27.52 (2.63)	22.65 (2.94)	<0.001
Neurodegenerative Risk/Disease	APOE 4 (n)	92 (valid n=285)	73 (valid n=159)	27 (valid n=45)	<0.001
	Tau: Meta Temporal Cortex (SUVR)	1.103 (0.110)	1.159 (0.151)	1.324 (0.264)	<0.001
	Amyloid: Early Amyloid Composite (SUVR)	1.096 (0.194)	1.201 (0.268)	1.430 (0.278)	<0.001

**Table 3.** Mean absolute error (MAE) for WM regional brain ages.

UKBB	Healthy L			Healthy R		
MAE	jxWM	dWM	pvWM	jxWM	dWM	pvWM
**Frontal**	2.20 (0.11)	2.24 (0.042)	2.46 (0.25)	2.21 (0.17)	2.47 (0.26)	2.27 (0.20)
**Occipital**	1.97 (0.17)	1.83 (0.15)	1.76 (0.27)	2.01 (0.12)	2.30 (0.15)	1.76 (0.24)
**Temporal**	2.41 (0.081)	2.43 (0.14)	2.39 (0.22)	2.43 (0.18)	2.35 (0.12)	1.96 (0.17)
**Parietal**	2.04 (0.34)	2.39 (0.17)	1.86 (0.12)	2.30 (0.14)	2.28 (0.15)	2.10 (0.15)
**Mean**		2.17 (0.25)			2.20 (0.20)	
